# NT157 has antineoplastic effects and inhibits IRS1/2 and STAT3/5 in JAK2^V617F^-positive myeloproliferative neoplasm cells

**DOI:** 10.1038/s41392-019-0102-5

**Published:** 2020-01-24

**Authors:** Bruna Alves Fenerich, Jaqueline Cristina Fernandes, Ana Paula Nunes Rodrigues Alves, Juan Luiz Coelho-Silva, Renata Scopim-Ribeiro, Priscila Santos Scheucher, Christopher A. Eide, Cristina E. Tognon, Brian J. Druker, Eduardo Magalhães Rego, João Agostinho Machado-Neto, Fabiola Traina

**Affiliations:** 1 Department of Medical Images, Hematology, and Clinical Oncology, University of São Paulo at Ribeirão Preto Medical School, Ribeirão Preto, São Paulo Brazil; 2 Center for Cell-Based Therapy, Sao Paulo Research Foundation, Ribeirão Preto, São Paulo Brazil; 3 Knight Cancer Institute, Oregon Health & Science University, Portland, OR USA; 4 Howard Hughes Medical Institute, Portland, OR USA; 5 Present Address: Department of Internal Medicine, University of São Paulo Medical School, São Paulo, Brazil; 6 Present Address: Department of Pharmacology, Institute of Biomedical Sciences of the University of São Paulo, São Paulo, Brazil

**Keywords:** Preclinical research, Haematological cancer

## Abstract

Recent data indicate that IGF1R/IRS signaling is a potential therapeutic target in BCR-ABL1-negative myeloproliferative neoplasms (MPN); in this pathway, IRS2 is involved in the malignant transformation induced by JAK2^V617F^, and upregulation of IGF1R signaling induces the MPN phenotype. NT157, a synthetic compound designed as an IGF1R-IRS1/2 inhibitor, has been shown to induce antineoplastic effects in solid tumors. Herein, we aimed to characterize the molecular and cellular effects of NT157 in JAK2^V617F^-positive MPN cell lines (HEL and SET2) and primary patient hematopoietic cells. In JAK2^V617F^ cell lines, NT157 decreased cell viability, clonogenicity, and cell proliferation, resulting in increases in apoptosis and cell cycle arrest in the G_2_/M phase (*p* < 0.05). NT157 treatment inhibited IRS1/2, JAK2/STAT, and NFκB signaling, and it activated the AP-1 complex, downregulated four oncogenes (*CCND1*, *MYB*, *WT1*, and *NFKB1*), and upregulated three apoptotic-related genes (*CDKN1A*, *FOS*, and *JUN*) (*p* < 0.05). NT157 induced genotoxic stress in a JAK2/STAT-independent manner. NT157 inhibited erythropoietin-independent colony formation in cells from polycythemia vera patients (*p* < 0.05). These findings further elucidate the mechanism of NT157 action in a MPN context and suggest that targeting IRS1/2 proteins may represent a promising therapeutic strategy for MPN.

## Introduction

Aberrant JAK/STAT signaling is a common feature in patients with Philadelphia chromosome-negative myeloproliferative neoplasm (MPN).^1^ The V617F point mutation in JAK2 kinase occurs in more than 90% of patients with polycythemia vera (PV) and in more than 50% of patients with essential thrombocythemia and primary myelofibrosis (PMF).^1,2^ Constitutive activation of the JAK2/STAT pathway results in dysregulation of critical processes involved in hematopoiesis, including apoptosis, proliferation, and differentiation.^3,4^ Cells harboring the JAK2^V617F^ mutation become hypersensitive to or independent of cytokines and growth factors, promoting proliferation, resulting in neoplastic overproduction of one or more lineages in the peripheral blood.^5^

Given its frequently mutated status and role in MPN pathogenesis, JAK2 represents a central target in MPN therapy.^6^ This spurred the development of selective JAK2 inhibitors such as ruxolitinib, a JAK1/2 inhibitor, which is approved for the treatment of patients with PMF or PV.^7^ However, despite this drug promoting clinical benefits, bone marrow fibrosis and malignant clones are not eradicated with therapy.^8–11^ The absence of complete clinical responses to JAK2 inhibitors in MPN patients is a matter of debate, prompting the study of proteins or signaling pathways that may contribute to mutated JAK2 signaling in MPN pathogenesis or to disease persistence despite pharmacological inhibition of JAK2.

In this scenario, proteins involved in cell proliferation and survival or those that connect important cytokines and growth factors involved in hematopoiesis may play a potential role in JAK2^V617F^-positive MPN. In previous studies, our research group focused on understanding the involvement of insulin receptor substrate (IRS) proteins in myeloid neoplasms.^12,13^ This family includes adaptor proteins that are activated by receptors involved in hematopoietic signaling, including IGF1R, EPOR, and TPOR (MPL).^14–16^ The interaction between the IRS1/2 and JAK2 proteins was initially demonstrated in cardiomyocytes and hepatocytes from rats upon angiotensin and leptin stimulation, respectively.^17,18^ Subsequent evidence indicates that IRS2 cooperates with malignant transformations induced by JAK2 mutations: (i) IRS2 binds to JAK2 in the HEL cell line model that harbors JAK2^V617F^ mutation, but it does not in the U937 JAK2 wild-type model lacking JAK/STAT signaling activation; (ii) IRS2 lentiviral-mediated silencing decreases STAT5 activation and cell viability and potentiates ruxolitinib-induced apoptosis in HEL cells.^13^ IRS2 mutations were described in triple-negative MPN patients.^19^ Osorio et al.^20^ demonstrated that IGFIR deregulation promotes MPN, incorporating new evidence that the IGF1R/IRS axis is potentially involved in the MPN phenotype and may represent a therapeutic target for these diseases.

The small-molecule inhibitor NT157 has been previously reported to promote IGF1R/IRS pathway inhibition through proteasomal degradation of IRS1/2 and antineoplastic activity in solid tumors.^21–26^ In the present study, we used this molecule to inhibit IRS2, a key adaptor protein in JAK2 (refs. ^17,18,27^) and IGF1R^28,29^ signaling, and characterized the efficacy of NT157 treatment in the context of JAK2^V617F^-positive MPN.

## Results

### NT157 treatment reduces viability and increases apoptosis in HEL cells

**Fig. 1:**
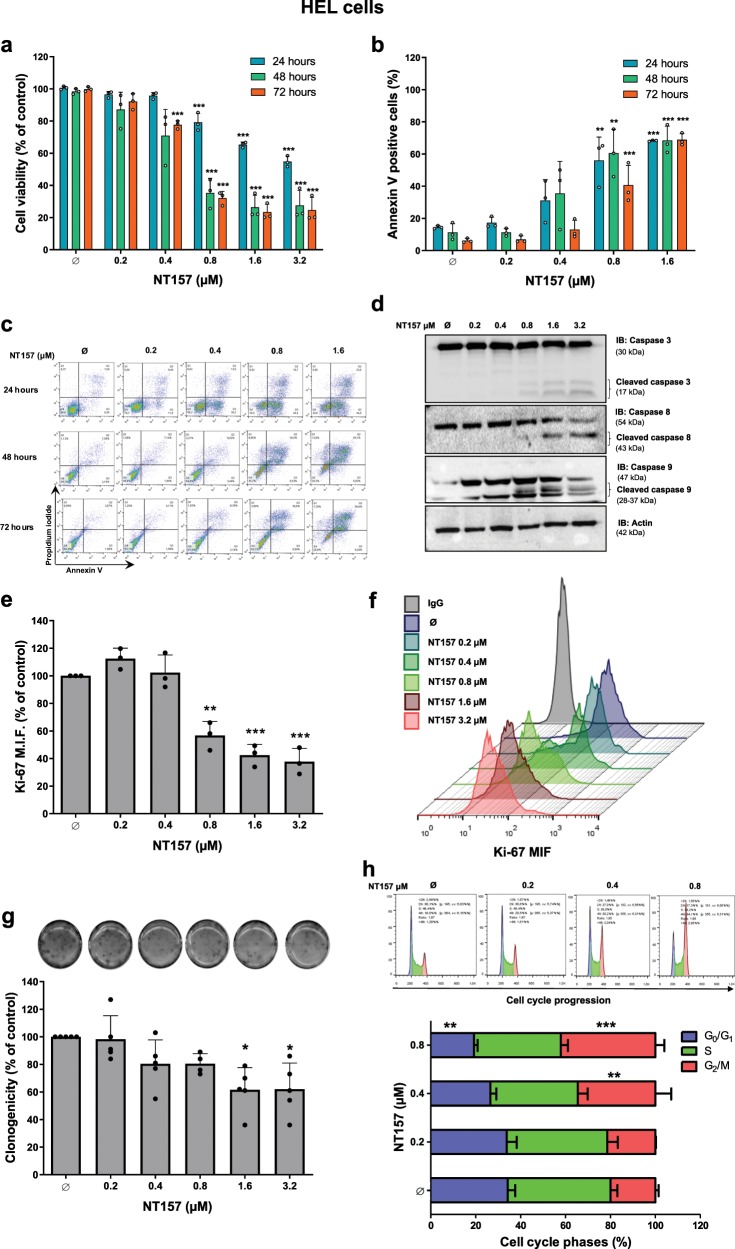
NT157 treatment results in a reduction in cell viability, proliferation, and clonogenicity and promotes induced mitotic catastrophe in HEL JAK2^V617F^ cells. **a** Cell viability was determined by methylthiazole tetrazolium (MTT) assays for HEL cells with or without NT157 treatment (0.2, 0.4, 0.8, 1.6, and 3.2 µM) for 24, 48, and 72 h. Bar graphs represent the mean ± SD of at least three independent experiments, and the dots represent the value of each experiment; ***p < 0.0001 for NT157-treated cells vs. untreated cells, and analysis was performed with an ANOVA test and a Bonferroni post-test. **b** Apoptosis was detected by flow cytometry in HEL cells with or without NT157 treatment (0.2, 0.4, 0.8, and1.6 µM) for 24, 48, and 72 h using an annexin V/PI staining method. Bar graphs represent the mean ± SD of at least three independent experiments where annexin V-positive cells were quantified, and the dots represent the value of each experiment; **p < 0.001 and *** *p* < 0.0001 for NT157-treated cells vs. untreated cells, and analysis was performed with an ANOVA test and a Bonferroni post-test. **c** Representative flow cytometry dot plots are shown for each condition; the upper and lower right quadrants cumulatively contain the apoptotic population (annexin V-positive cells). **d** Caspases 3, 8, and 9 levels (total and cleaved) were assessed by western blot analysis in total cell extracts from HEL with NT157 treatment (Ø, 0.2, 0.4, 0.8, 1.6, and 3.2 µM) for 24 h; membranes were reprobed with antibodies to detect total target protein and/or actin, and then they were developed with a SuperSignal™ West Dura Extended Duration Substrate system and a Gel Doc XR + system. **e** Ki-67 mean fluorescence intensity (MFI) was determined by flow cytometry after incubation of HEL cells with NT157 (Ø, 0.2, 0.4, 0.8, 1.6, and 3.2 µM) for 24 h; bar graphs represent the Ki-67 MFI normalized to the respective untreated control cells, and the results are shown as the mean ± SD of three independent experiments. The dots represent the value of each experiment; **p < 0.001, and ***p < 0.0001, and analysis was performed with an ANOVA test and a Bonferroni post-test. **f** Histograms show Ki-67 MFI peaks for all tested conditions. Each condition is represented by one color that is defined in the legend located in the left panel. **g** Colonies containing viable cells were detected by MTT assay after 10 days of culture with NT157, and data are normalized to the corresponding untreated control. Colony images are shown for one experiment, and the bar graphs show the mean ± SD of five independent experiments. The dots represent the value of each experiment; *p < 0.05 for NT157-treated cells vs. untreated cells, and analysis was performed with an ANOVA test and a Bonferroni post-test. **h** Cell cycle progression was determined by a BD Cycletest™ Plus DNA Reagent Kit in HEL cells treated with NT157 (Ø, 0.2, 0.4, and 0.8 µM) for 24 h. A representative histogram for each condition is illustrated; cell cycle phases are detected based on DNA quantity: blue (cells in G_0_/G_1_), green (cells in S), and pink (G_2_/M cells). Bar graphs represent the mean ± SD of the percent of cells in the G_0_/G_1_, S, and G_2_/M phase of three independent experiments; **p < 0.001 and ***p < 0.0001, and analysis was performed with an ANOVA test and a Bonferroni post-test.

We first evaluated the effects of NT157 treatment on cell viability and apoptosis in the JAK2^V617F^-positive human leukemia cell line HEL. The IC_50_ values for NT157 in HEL cells were 3.1, 0.68, and 0.72 µM at 24, 48, and 72 h, respectively. Treatment with NT157 at concentrations at or above 0.8 μM significantly reduced cell viability (Fig. 1a) and increased apoptosis in a dose-dependent manner (all *p* < 0.001) (Fig. 1b, c). To validate the data obtained by flow cytometry, we evaluated caspase cleavage after 24 h of exposure to different concentrations of NT157. Treatment with NT157 increased levels of cleaved caspases 3, 8, and 9 in a dose-dependent manner (Fig. 1d).

### NT157 reduces proliferation and clonogenicity in HEL cells and induces mitotic catastrophe

The effect of NT157 on cell proliferation was assessed by Ki-67 staining. NT157 treatment reduced cell proliferation in a dose-dependent manner; the number of proliferating cells was reduced by 43, 58, and 62% at doses of 0.8, 1.6, and 3.2 μM, respectively (*p* < 0.0001) (Fig. 1e, f). Long-term exposure to 3.2 μM NT157 significantly reduced clonogenic capacity (Fig. 1g). To understand the mechanisms involved in cell growth inhibition, cell cycle progression was evaluated 24 h after NT157 treatment. NT157 promoted G_2_/M accumulation, and there was an associated decrease in the G_0_/G_1_ population (*p* < 0.001) (Fig. 1h; Supplementary Table S1).

### NT157 inhibits JAK2/STAT signals in a phosphatase-dependent manner

**Fig. 2:**
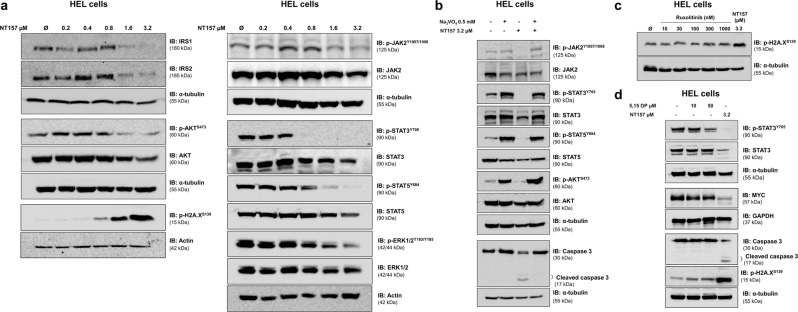
NT157 downregulates IRS1/2, JAK2/STAT, AKT, and ERK1/2 signaling and induces DNA damage in HEL cells. **a** Western blot analysis is shown for IRS1, IRS2, p-AKT^S473^, AKT, p-H2A.X^S139^, p-JAK2^T1007/1008^, JAK2, p-STAT3^Y705^, STAT3, p-STAT5^Y694^, STAT5, p-ERK1/2^T183/Y185^, and ERK1/2 levels in total cell extracts from HEL cells treated with NT157 (Ø, 0.2, 0.4, 0.8, 1.6, and 3.2 µM) for 24 h. **b** Western blot analysis is shown for p-JAK2^T1007/1008^, JAK2, p-STAT3^Y705^, STAT3, p-STAT5^Y694^, STAT5, p-AKT^S473^, AKT, and caspase 3 levels in total cell extracts from HEL cells previously treated with Na_3_VO_4_ (0.5 mM) for 30 min followed by NT157 (3.2 µM) treatment for an additional 24 h as indicated. **c** Western blot analysis is shown for p-H2A.X^S139^ from HEL cells treated with ruxolitinib (Ø, 10, 30, 100, 300, and 1000 nM) or NT157 3.2 µM for 24 h. **d** Western blot analysis is shown for p-STAT3^Y705^, STAT3, MYC, caspase 3, and p-H2A.X^S139^ levels in total cell extracts from HEL cells treated with 5,15-DP (Ø, 10 and 50 µM) or NT157 (3.2 µM) for 24 h. Membranes were reprobed with an antibody for the detection of total target protein and/or actin, GAPDH or α-tubulin, and images were detected with a SuperSignal™ West Dura Extended Duration Substrate system using a Gel Doc XR+ imaging system.

We next explored the molecular effects of NT157 on proteins involved in intracellular signaling. Western blot analysis revealed that NT157 (1.6 and 3.2 μM) was able to reduce total levels of IRS1 and IRS2, inhibit JAK2, STAT3, STAT5, AKT, and ERK1/2 phosphorylation and induce DNA damage and mitotic catastrophe, as demonstrated by increased H2AX phosphorylation; further, these effects occurred in a dose-dependent manner (Fig. 2a). Phosphatase inhibition with Na_3_VO_4_ prevented JAK2, STAT3, STAT5, and AKT dephosphorylation and caspase 3 cleavage by NT157 (Fig. 2b). H2AX phosphorylation was not induced by pharmacological inhibition of JAK2/STAT with ruxolitinib or by treatment with a selective STAT3-SH2 antagonist (5,15-DPP), indicating that NT157-induced genotoxic stress was independent of JAK2/STAT inhibition (Fig. 2c, d). Of note, the selective STAT3-SH2 antagonist was able to inhibit STAT3 phosphorylation and MYC expression, but it was not able to induce caspase 3 cleavage (Fig. 2d) or decrease HEL cell viability (Supplementary Fig. S1).

### Treatment with NT157 modulates the expression of oncogenes and tumor suppressor genes in HEL cells

**Fig. 3:**
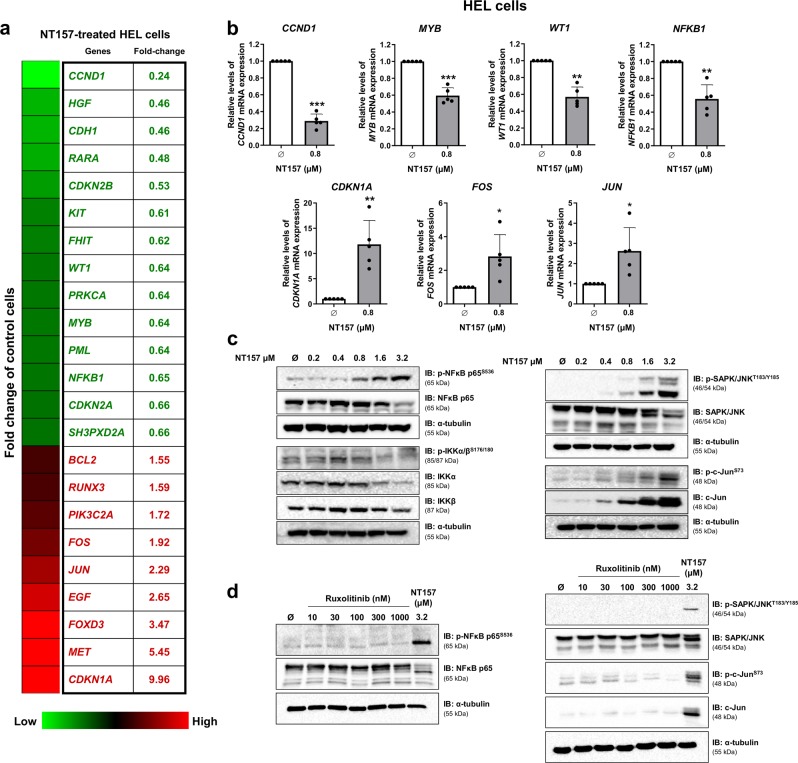
NT157 modulates oncogenes and tumor suppressor genes and signaling, including NFκB downregulation and JNK signaling activation, in HEL cells. **a** Gene expression is illustrated with a heatmap from PCR array analysis of HEL cells treated with NT157 (0.8 µM) for 16 h. mRNA levels are normalized to those of untreated HEL cells and are calculated as fold change in expression; genes presenting ≥1.5-fold in either direction compared to untreated cells are included in the heatmap. Two independent experiments of each condition were used for the analysis; green indicates repressed mRNA levels, and red indicates elevated mRNA levels. **b** Validation of seven genes involved in cell cycle progression and apoptosis in five independent experiments: qPCR analysis is shown for *CCND1, MYB, WT1, NFKB1, CDKN1A, FOS*, and *JUN* expression in HEL cells treated with NT157 (0.8 µM) for 16 h. Bar graphs represent the mean ± SD of at least five independent experiments, and the dots represent the value of each experiment; *p < 0.05, ** *p* < 0.001, and ***p < 0.0001, and analysis was performed with a Student's *t-*test. **c** Western blot analysis is shown for p-NFκB p65^S536^, NFκB p65, p-IKKα/β^S176/180^, IKKα, IKKβ, p-SAPK/JNK^T183Y185^, SAPK/JNK, p-c-Jun^S73^, and c-Jun in total cell extracts from HEL cells treated with NT157 (Ø, 0.2, 0.4, 0.8, 1.6, and 3.2 µM) for 24 h. **d** Western blot analysis is shown for p-NFκB p65^S536^, NFκB p65, p-SAPK/JNK^T183Y185^, SAPK/JNK, p-c-Jun^S73^, and c-Jun from HEL cells treated with ruxolitinib (Ø, 10, 30, 100, 300, and 1000 nM) or NT157 3.2 µM for 24 h. Membranes were reprobed with an antibody to detect the target total protein and/or α-tubulin, and images were then developed with a SuperSignal™ West Dura Extended Duration Substrate system using a Gel Doc XR+ imaging system.

To identify additional molecular targets of NT157, we evaluated the expression profile of a panel of 84 key genes in the neoplastic process by PCR array. Treatment with NT157 (0.8 μM) for 16 h modulated mRNA expression levels of 23 genes by ≥1.5-fold in either direction compared to untreated cells: 14 downregulated genes (*CCND1*, *HGF*, *CDH1*, *RARA*, *CDKN2B*, *KIT*, *FHIT*, *WT1*, *PRKCA*, *MYB*, *PML*, *NFKB1*, *CDKN2A*, and *SH3PXD2A*) and 9 upregulated genes (*BCL2*, *RUNX3, PIK3C2A, FOS, JUN, EGF, FOXD3, MET*, and *CDKN1A*) (Fig. 3a, Supplementary Table S2). Based on the cellular effects observed after NT157 treatment and the description of these genes in hematologic neoplasms, four repressed oncogenes (*CCND1*, *MYB, WT1*, and *NFKB1*) and three upregulated apoptosis-related genes (*CDKN1A*, *FOS*, and *JUN*) were selected and validated in a larger number of experiments (*n* = 5) (all *p* < 0.05) (Fig. 3b). Downregulation of NFκB-mediated signaling and upregulation of JNK-mediated signaling upon NT157 treatment was confirmed by western blotting analysis (Fig. 3c), and they were independent of JAK2/STAT inhibition (Fig. 3d).

### Combined treatment with NT157 and ruxolitinib does not enhance apoptosis and cell cycle arrest beyond the results from treatment with single agents

**Fig. 4:**
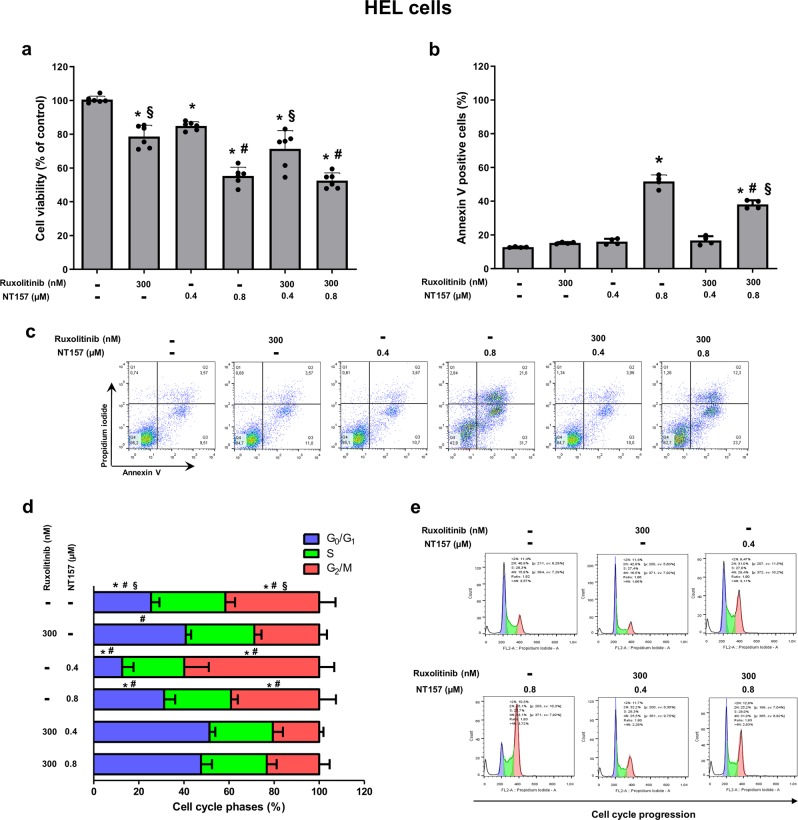
The antineoplastic effects of NT157 are not enhanced by combined treatment with ruxolitinib in HEL cells. **a** Cell viability was determined by MTT assay for HEL cells treated with a monotherapy and a combined therapy in different conditions for 48 h. Bar graphs represent the mean ± SD of six independent experiments; the dots represent the value of each experiment. **b** Apoptosis was detected by flow cytometry using an annexin V/PI staining method in HEL cells treated under the same conditions described for the MTT assay. Bar graphs represent the mean ± SD of at least four independent experiments that quantified apoptotic cell death; the dots represent the value of each experiment. **c** Representative flow cytometry dot plots are shown for each condition; the upper and lower right quadrants cumulatively contain the apoptotic population (annexin V-positive cells). **d** Cell cycle progression was determined by a BD Cycletest™ Plus DNA Reagent Kit in HEL cells treated with a monotherapy and a combined therapy in different conditions for 24 h. Bar graphs represent the mean ± SD of the percent of cells in each phase. For all graphics presented, *p < 0.05 related to untreated cells, ^#^*p* < 0.05 related to ruxolitinib monotherapy, and ^§^*p* < 0.05 related to NT157 monotherapy in the corresponding dose; analyses were performed with ANOVA tests and Bonferroni post-tests. **e** A representative histogram for each condition is shown; cell cycle phases are detected based on DNA quantity: blue (cells in G_0_/G_1_), green (cells in S), and pink (G_2_/M cells).

To compare the side-by-side effects of NT157 with the prototype JAK1/2 inhibitor ruxolitinib and to verify the additional or differential effects of both compounds on cell viability, apoptosis and cell cycle progression, we tested the biological effects of NT157 and ruxolitinib as monotherapies and a cotreatment. Doses of NT157 and ruxolitinib were selected according to IC_50_ values for each inhibitor alone. The ruxolitinib dose was determined by a dose–response curve that was evaluated by western blot; 300 nM was selected, since it was the minimal dose that completely inhibited STAT3 and STAT5 phosphorylation (Supplementary Fig. S2). HEL cells were treated for 48 h and submitted to viability, apoptosis, and cell cycle assays. In HEL cells, both monotherapies were capable of reducing cell viability compared to untreated cells (*p* < 0.05). However, when combined (0.8 µM NT157 plus 300 nM ruxolitinib), the effect was not significantly different from the outcome observed when treating with NT157 alone (Fig. 4a). Potential synergy was also tested using an expanded six-dose curve for each drug, with combination index (CI) values ranging from synergistic to additive and even antagonist (Supplementary Fig. S3a). By this metric, the combination of 300 nM ruxolitinib plus 0.4 or 0.8 µM NT157 was classified as synergistic or moderately synergistic (CI = 0.59 and 0.64, respectively). In contrast, NT157 treatment resulted in slightly enhanced apoptosis (52%) compared to the combination of NT157 plus ruxolitinib (38%) (*p* < 0.05) (Fig. 4b, c). Ruxolitinib also prevented NT157-induced G_2_/M arrest in HEL cells (*p* < 0.05) (Fig. 4d, e; Supplementary Table S3).

### NT157 treatment promotes antineoplastic effects in SET2 at higher doses

**Fig. 5:**
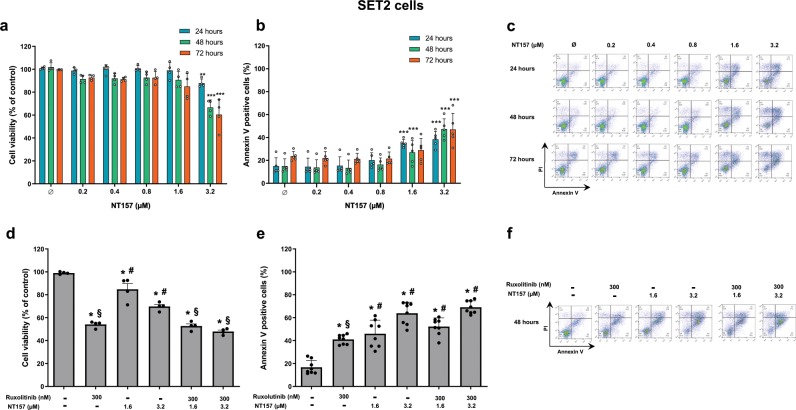
In SET2^V617F^ cells, high doses of NT157 produce antineoplastic effects, but combined NT157 and ruxolitinib treatment does not potentiate the effects of monotherapies. **a** Cell viability was determined by methylthiazole tetrazolium (MTT) assay for SET2 cells with or without NT157 treatment (0.2, 0.4, 0.8, 1.6, and 3.2 µM) for 24, 48, and 72 h. Bar graphs represent the mean ± SD of four independent experiments, and the dots represent the value of each experiment; **p < 0.001, and ***p < 0.0001 for NT157-treated cells vs. untreated cells, and analysis was performed with an ANOVA test and a Bonferroni post-test. **b** Apoptosis was detected by flow cytometry in SET2 cells with or without NT157 treatment (0.2, 0.4, 0.8, 1.6, and 3.2 µM) for 24, 48, and 72 h using an annexin V/PI staining method. Bar graphs represent the mean ± SD of five independent experiments quantifying apoptotic cell death; the dots represent the value of each experiment. **c** Representative flow cytometry dot plots are shown for each condition. **d** Cell viability was determined by MTT assay for SET2 cells treated with NT157 and/or ruxolitinib for 48 h. Bar graphs represent the mean ± SD of four independent experiments; the dots represent the value of each experiment. **e** Apoptosis was detected by flow cytometry in SET2 cells treated under the same conditions described for the MTT assay using an annexin V/PI staining method. Bar graphs represent the mean ± SD of eight independent experiments quantifying apoptotic cell death. For all graphs presented, *p < 0.05 related to untreated cells, ^#^*p* < 0.05 related to ruxolitinib monotherapy, and ^§^*p* < 0.05 related to NT157 monotherapy in the corresponding dose; analyses were performed with ANOVA tests and Bonferroni post-tests. **f** Representative flow cytometry dot plots are shown for each condition.

To provide further evidence and a point of comparison for the results observed in HEL cells, viability and apoptosis analyses were expanded to JAK2^V617F^-positive SET2 cells. At the highest NT157 concentration tested (3.2 µM), cell viability was significantly reduced (*p* ≤ 0.05) (Fig. 5a) in conjunction with a significant induction of apoptosis (*p* < 0.001) (Fig. 5b, c). Ruxolitinib (300 nM) showed a greater effect on reducing cell viability than NT157 (*p* < 0.001), and the combined treatment did not significantly enhance the efficacy beyond what was observed when treating with ruxolitinib alone (Fig. 5d). In contrast, NT157 treatment showed a greater effect on apoptosis induction than ruxolitinib treatment (Fig. 5e, f), and the effect of a combined treatment was not significantly more effective than treatment with NT157 alone. Synergism calculations similar to those performed for HEL cells showed that ruxolitinib (300 nM) plus NT157 (0.4 or 0.8 µM) was synergistic (CI = 0.48 and 0.46, respectively) (Supplementary Fig. S3b), though this effect was not observed in the functional assays.

### NT157 inhibits erythropoietin-independent colony formation in cells from polycythemia vera patients

**Fig. 6:**
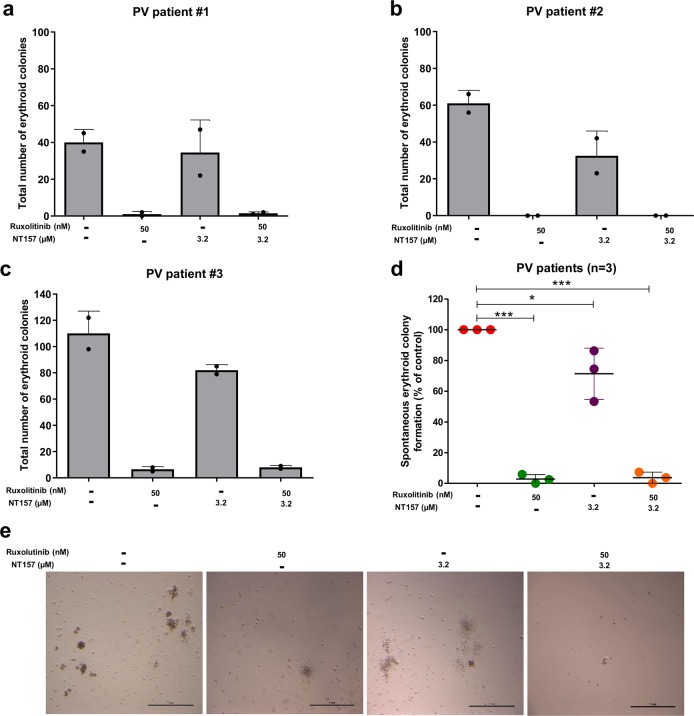
NT157 reduces spontaneous erythroid colony formation in primary polycythemia vera cells. **a–c** Peripheral blood (patient #1 and #2) or bone marrow (patient #3) mononuclear cells from polycythemia vera (PV) patients were plated on methylcellulose containing cytokines but lacking erythropoietin in the presence or absence of NT157 (3.2 µM) and/or ruxolitinib (50 nM). Spontaneously formed erythroid colonies were counted after 14 days of culture and are represented as the total number produce under each condition. Bars indicate the mean ± SD of the duplicate assays for each patient; the dots represent the value of each duplicate. **d** Dot plot comparing combined colony formation results from all three PV patients. The horizontal line represents the mean ± SD. The *p* values are indicated in the graphs; *p < 0.05, and ***p < 0.0001 for NT157 and/or ruxolitinib-treated cells vs. untreated controls, and analysis was performed with an ANOVA test and a Bonferroni post-test. **e** Representative images of erythropoietin-independent colony formation at 14 days of culture from one PV patient are illustrated.

NT157 (3.2 μM) treatment significantly reduced erythropoietin-independent colony formation in peripheral (*n* = 2) and bone marrow (*n* = 1) mononuclear cells from PV patients by an average of 29% (*p* < 0.05), and the range was 14–47%. As a positive control, we used ruxolitinib (50 nM) at a dose capable of strongly reducing erythropoietin-independent colony formation (Fig. 6).

## Discussion

Previous studies from our research group demonstrated that IRS2 associates with JAK2 in HEL JAK2^V617F^ cells,^13^ pointing to IRS2 as a novel, potential target in JAK2^V617F^-positive MPN. To target IRS2 in the present study, we utilized the small-molecule inhibitor NT157, which was developed for long-term inhibition of this pathway through proteasome-mediated IRS1/2 degradation.^26^ We found that in the JAK2^V617F^ cell line, treatment with NT157 inhibited IRS1/2 and STAT3/5 signaling and resulted in remarkable antineoplastic activity, including a decrease in cell viability, proliferation, and clonogenicity and was accompanied by an induction of apoptosis and mitotic catastrophe. NT157 was also effective in inhibiting erythropoietin-independent colony formation in samples from PV patients.

Exposure to NT157 resulted in decreased ERK1/2 phosphorylation in HEL cells. Reuveni et al.^26^ found increased ERK/MAPK activation after exposure to this compound and showed that IRS1/2 degradation is mediated by this pathway. Our results indicate that ERK1/2 modulation upon NT157 treatment may be cell line- and time-dependent.

STAT3 inhibition upon NT157 treatment has also been described in other solid tumor cell lines and in multiple myeloma.^23,24^ Flashner-Abramson et al.^23^ described that in a melanoma cell line, NT157 inhibited STAT3 in a phosphatase-dependent and IGF1R/IRS-independent manner. In HEL cells, NT157 inhibited JAK2/STAT in a phosphatase-dependent manner. Intriguingly, we observed that NT157 treatment presents the advantage of modulating the phosphorylation of not only STAT3 but also STAT5, which is more directly related to myeloid expansion induced by JAK2^V617F^.^30^ In Ba/F3 cells, it has been shown that constitutive activation of STAT5 is required for neoplastic transformation mediated by JAK2^V617F^.^31^ In vitro, pharmacological inhibition of STAT5 using pimozide potentiated the effects of a JAK2 inhibitor, increasing death in HEL, SET2, and Ba/F3 JAK2^V617F^ cells.^32^ Corroborating these findings, Yan et al.^33^ demonstrated in vivo that Stat5 is required for and plays a critical role in PV development in a murine MPN model. These data reinforce the therapeutic potential of STAT5 inhibition in JAK2^V617F^-driven MPN. Notably, we previously observed that lentivirus-mediated silencing of IRS2 inhibited STAT5 activation in HEL cells,^13^ raising the possibility that in this cell line model, STAT5 inhibition may be either a consequence of IRS2 inhibition or a direct result of the action of the inhibitor.

STAT5 regulates oncogenic signaling, including survival and cell cycle progression in the neoplastic clone.^34^ Therefore, STAT5 inhibition may explain one of the mechanisms responsible for the effects elicited by NT157 in HEL cells. Upon NT157 treatment, HEL cells presented decreased cell proliferation associated with downregulation of *CCND1* expression. Decreased levels of this oncogene are in accordance with the reduction of STAT3 and STAT5 phosphorylation, since both proteins positively regulate the expression of *CCND1*.^35,36^

NT157 induced a DNA damage response that was independent of JAK2/STAT signaling, a delay in cell cycle progression in G_2_/M, and an increase in apoptosis. Consistent with this, we observed increased *CDKN1A* (p21), which is a tumor suppressor gene mainly expressed in the G_2_/M phase under p53 protein control during the cellular checkpoint for DNA damage.^37^ In a broad spectrum of tumors, including hematological malignancies, CDKN1A is repressed and is associated with poor prognosis.^38^ Reduction of *MYB* levels may also contribute to the cell cycle arrest observed in HEL cells, given its role in the G_2_/M transition through direct regulation of cyclin B1 expression in normal and neoplastic hematopoietic cells.^39^

Increased apoptosis induced by NT157 may also be correlated with repression of the WT1 oncogene and upregulation of apoptotic-related genes, including FOS and JUN. High expression of WT1 is related to proliferative alteration, increased numbers of blasts, progression to AML and repression of pro-apoptotic genes, such as BAK, which in turn contributes to the survival of neoplastic cells.^40,41^ NT157 treatment resulted in increased FOS and JUN gene expression and JNK activation. FOS dimerizes with the transcription factor JUN to form the AP-1 complex, which controls the expression of multiple genes involved in cell proliferation, apoptosis, and differentiation.^42^ The activation of JNK protein kinase can result in the phosphorylation of the AP-1 complex, mediating apoptosis induced by cellular stress.^43^ This hypothesis is consistent with increased JNK activation in melanoma cells upon NT157 treatment.^44^ NFκB activation has been associated with resistance to apoptosis and uncontrolled proliferation^45^ in myeloid neoplasms, and targeting NFκB-mediated signaling attenuated the MPN-related phenotype in JAK2^V617F^ mice model.^46^ Thus, NFκB downregulation mediated by NT157, as observed by NFκB p65 phosphorylation at serine 536, an inhibitory site,^47^ and reduced IKKα/β activation may contribute to cytotoxicity of the drug.

In the present study, ruxolitinib was employed as a JAK2/STAT inhibitor, which served as a positive control, since its mechanism of action is well elucidated. It targets the pivotal pathway affected in MPN, and it is FDA approved for the treatment of PV and PMF. Although the comparison is experimentally expected, it is noteworthy that the study was not conducted to propose a combined therapy. In JAK2^V617F^ cell line models, the antineoplastic activity of NT157 was not enhanced by the combined treatment. Indeed, the effects of combined treatment appeared mainly cytostatic and actually dampened, at least in part, the cytotoxic effects of NT157 in HEL cells. Similar findings were observed in SET2 cells; however, SET2 cells were less sensitive to NT157 than HEL cells.

In summary, NT157 exerts antineoplastic effects in JAK2^V617F^-positive cells by targeting many mechanisms, downregulating IRS1/2, JAK2/STAT, NFκB signaling, and activating the AP-1 complex in MPN. Our findings further highlight IRS2 as a therapeutic target and provide new insights into the molecular mechanisms of NT157 in JAK2^V617F^ mutant MPN.

## Material and methods

### Cell lines, pharmacological inhibitors, and treatment strategy

Human erythroleukemia HEL 92.1.7 (HEL) (homozygous JAK2^V617F^) and SET2 (heterozygous JAK2^V617F^) cells were used. Cell lines were obtained and maintained for experiments as previously described.^48^ NT157 was kindly provided by Dr. Reuveni et al.^26^ for initial testing and was subsequently acquired from Sun-Shinechem (Sun-Shinechem, Wuhan, China). Ruxolitinib was obtained from InvivoGen (San Diego, CA, USA). NT157 and ruxolitinib were dissolved in dimethyl sulfoxide (DMSO) (Sigma-Aldrich, Missouri, USA) and stored in stock solutions of 10 and 20 mM, respectively (final concentration of DMSO was less than 0.003% by volume). Cell lines were exposed to NT157 (0.2, 0.4, 0.8, 1.6, and 3.2 µM) for 24, 48, and 72 h and were analyzed by cell viability assays. IC_50_ values were calculated using CalcuSyn software (Biosoft, Ferguson, MO, USA). For synergism evaluation, cell lines were treated with different doses of ruxolitinib (3, 10, 30, 100, 300, 1000 nM) and/or NT157 (0.2, 0.4, 0.8, 1.6, 3.2 µM) for 48 h and submitted to a cell viability assay. CompuSyn software (ComboSyn, Inc., Paramus, NJ, USA) was applied for combination index (CI) calculation, and data were described according to Chou^49^ and illustrated using multiple experiment viewer (MeV) 4.9.0 software (http://www.tm4.org/mev/). For combined treatment, NT157 doses were chosen based on the CI and sensitivity of each cell line (0.4 and 0.8 for HEL cells; 1.6 and 3.2 µM for SET2 cells). Sodium orthovanadate (Na_3_VO_4_) (Sigma-Aldrich) was diluted in water and used at 0.5 mM as a phosphatase inhibitor. 5,15-DPP (5,15-diphenylporphyrin) (Sigma-Aldrich) was diluted in DMSO and was used at 10 and 50 µM concentrations as a selective STAT3-SH2 antagonist.^50^

### Functional studies

Cells were subjected to cell viability and apoptosis assays as previously reported.^48^ Briefly, after 4 h of serum deprivation, HEL and SET2 cells were seeded in 24-well plates and treated with NT157 (Ø, 0.2, 0.4, 0.8, 1.6, and 3.2 µM) for 24, 48, and 72 h. For combined treatment studies, cells were exposed to NT157 (0.4 and 0.8 µM for HEL cells; 1.6 and 3.2 µM for SET2 cells) and/or ruxolitinib (300 nM) for 48 h. Similarly, after 4 h of serum deprivation, HEL cells were treated with NT157 (Ø, 0.2, 0.4, 0.8, 1.6, and 3.2 µM) for 24 h, fixed with 70% ethanol, were stored at −20 °C, and then were subjected to cell proliferation analysis as measured by Ki-67 staining, according to previously described methods.^48^

Colony formation capacity was carried out in semisolid methylcellulose medium (MethoCult 4230; StemCell Technologies Inc., Vancouver, BC, Canada). HEL cells were seeded in the absence or presence of NT157 (0.2, 0.4, 0.8, 1.6, 3.2 µM), and colonies were quantified after 10 days of culture by adding 1 mg/mL of MTT reagent and were scored by ImageJ quantification software (U.S. National Institutes of Health, Bethesda, MD, USA).

HEL cells were cultured in the presence of NT157 (Ø, 0.2, 0.4, 0.8 µM) and/or ruxolitinib (300 nM) for 24 h, and their cell cycle evaluation was performed with a BD Cycletest™ Plus DNA Reagent Kit (Becton-Dickinson, Mountain View, CA, USA) as previously described.^48^ Doses above 0.8 µM were not included, as MTT and apoptosis assays revealed a significant number of dead cells at these concentrations.

### Protein and gene expression profile

Total protein extracts were analyzed by western blot as previously reported.^48^ Antibodies (Supplementary Table S4) and whole-gel images are shown (Supplementary Fig. S4).

Total RNA from HEL cells exposed to NT157 (0.8 µM; 16 h) was submitted for PCR array analysis (Human Oncogenes & Tumor Suppressor Genes RT² Profiler™ Kit [#PAHS-502Z, QIAGEN, Hilden, Germany]). Gene expression in treated cells was normalized to that of untreated cells, and a fold change ≥1.5-fold in any direction was included in the heatmap using heatmap builder software (The Ashley Lab, Stanford University, CA, USA).

Quantitative PCR with specific primers (Supplementary Table S5) for cyclin D1 (*CCND1*), Myb proto-oncogene (*MYB*), Wilms Tumor 1 (*WT1*), *nuclear factor kappa B subunit 1* (*NFKB1*), cyclin-dependent kinase inhibitor 1A (*CDKN1A*), JUN proto-oncogene (*JUN*), Fos proto-oncogene (*FOS*), hypoxanthine phosphoribosyltransferase 1 (*HPRT1*), glyceraldehyde 3-phosphate dehydrogenase (*GAPDH*), and/or beta actin (*ACTB*) were used for validation, as previously described.^48^

### Erythropoietin-independent colony formation

This research was approved by the Research Ethics Committee in Human Research at the General Hospital of University of São Paulo at Ribeirão Preto Medical School (CEP HC FMRP). Written informed consent was obtained from all subjects, and the study was performed in accordance with the Declaration of Helsinki. Peripheral blood (*n* = 2) or bone marrow (*n* = 1) mononuclear cells from PV patients were analyzed via erythropoietin-independent colony formation assay in the absence or presence of NT157 (3.2 µM) and/or ruxolitinib (50 nM), as previously described.^48^

### Statistical analysis

Statistical analyses were performed using GraphPad Prism 5 (GraphPad Software, Inc., San Diego, CA, USA). For comparisons, Student’s *t*-tests or ANOVA tests with Bonferroni post-test were used. At least three independent experiments for each method were tested. A *p* value < 0.05 was considered statistically significant.

## Supplementary information

Supplementary Material

Dataset 1

## Data Availability

The datasets used and/or analyzed during the current study are available from the corresponding author on reasonable request.
