# Supplementary material for: NT157 has antineoplastic effects and inhibits IRS1/2 and STAT3/5 in JAK2V617F-positive myeloproliferative neoplasm cells
Source: Signal Transduct Target Ther. 2020 Jan 24;5:5. doi: 10.1038/s41392-019-0102-5 (PMC6978524; doi:10.1038/s41392-019-0102-5)
Supplement: Supplementary file 1 — Supplementary Material [file 41392_2019_102_MOESM1_ESM.doc]

**Supplementary Information**

**NT157 has antineoplastic effects and inhibits IRS1/2 and STAT3/5 in JAK2^V617F^-positive myeloproliferative neoplasm cells**

Bruna Alves Fenerich^1,2^, Jaqueline Cristina Fernandes^1,2^, Ana Paula Nunes Rodrigues Alves^1,2^, Juan Luiz Coelho-Silva^1,2^, Renata Scopim-Ribeiro^1,2^, Priscila Santos Scheucher^1^, Christopher A. Eide^3,4^, Cristina E. Tognon^3,4^, Brian J. Druker^3,4^, Eduardo Magalhães Rego^1,2,5^, João Agostinho Machado-Neto^1,6^, Fabiola Traina^1,2^*

^1^Department of Medical Images, Hematology, and Clinical Oncology, University of São Paulo at Ribeirão Preto Medical School, Ribeirão Preto, São Paulo, Brazil

^2^Center for Cell-Based Therapy, Sao Paulo Research Foundation, Ribeirão Preto, SP, Brazil;

^3^Knight Cancer Institute, Oregon Health & Science University, Portland, Oregon, USA

^4^Howard Hughes Medical Institute, Portland, Oregon, USA

^5^Currently at Department of Internal Medicine, University of São Paulo Medical School, São Paulo, Brazil

^6^Currently at Department of Pharmacology, Institute of Biomedical Sciences of the University of São Paulo, São Paulo, Brazil

**This file includes:**

Supplementary Figures S1 to S4

Supplementary Tables S1 to S5

|  |
| --- |
| **Supplementary Figure S1. Selective STAT3-SH2 antagonist, 5,15DPP, did not modulates cell viability in HEL JAK2V617F cells.** Cell viability was determined by methylthiazoletetrazolium (MTT) assay for HEL cells treated or not with NT157 (10, 20, 50 and 100 μM) for 24 hours. Bar graphs represent the mean±SD of five independent experiments; the dots represent the value of each experiment. |

|  |
| --- |
| S**upplementary Figure S2. Ruxolitinib dose-effect curve on STAT3 and STAT5 activation in HEL cells**. Representative Western blot for the evaluation of the effects of different ruxolitinib concentrations on the activation of STAT proteins in total extracts of HEL cells treated with ruxolitinib (Ø, 10, 30, 100, 300 and 1000 nM) for 24 hours. Membranes were reprobed with the antibody for the detection of the respective total protein or actin and developed with the SuperSignal™ West Dura Extended Duration Substrate system and a Gel Doc XR+ imaging system. |

| 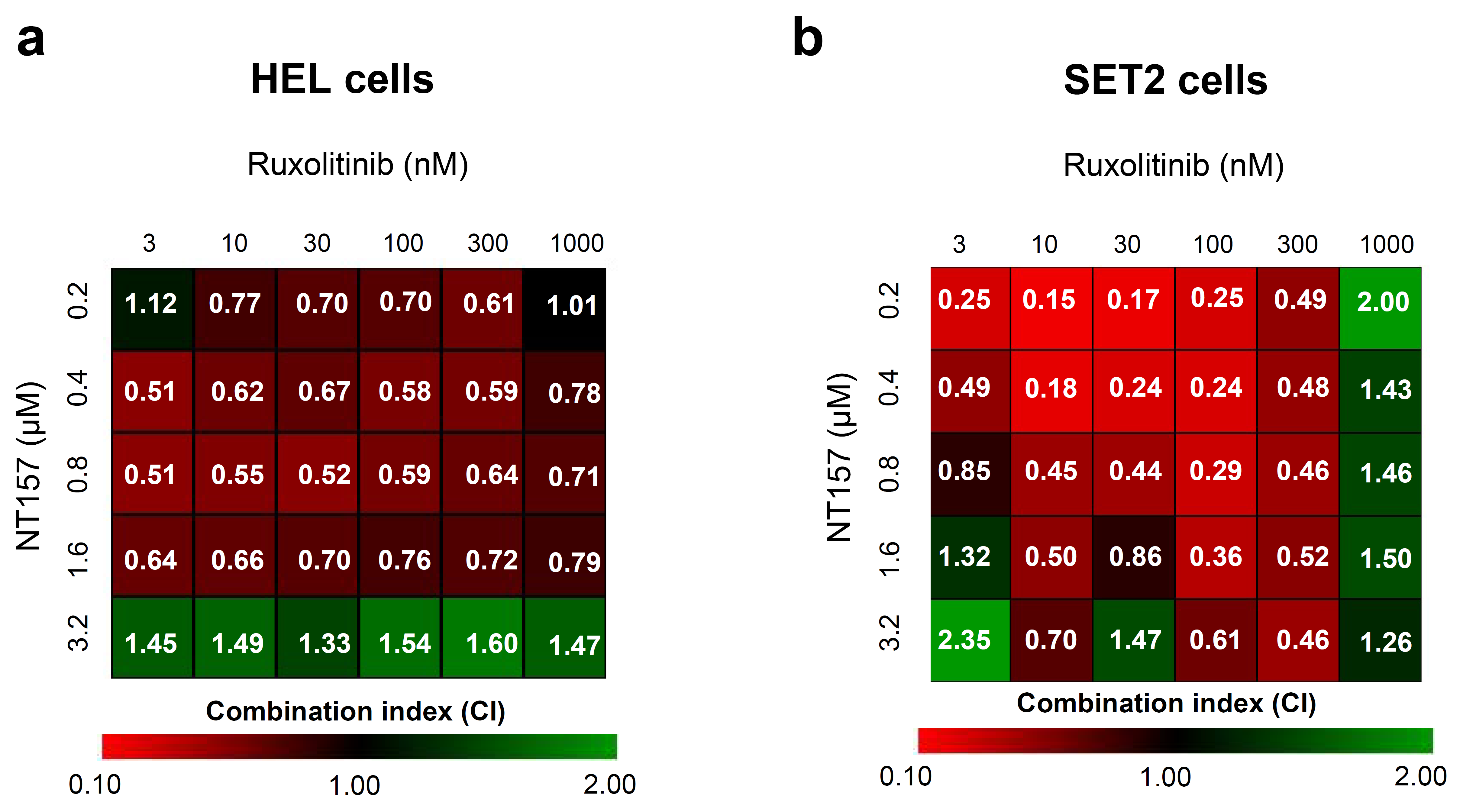 |
| --- |
| **Supplementary Figure S3. Combination index of ruxolitinib and NT157 in HEL and SET2 cells. (a)** HEL and **(b)** SET2 cells were treated with graded doses of ruxolitinib (3, 10, 30,100, 300 and 1000 nM) and NT157 (0.2, 0.4, 0.8, 1.6 and 3.2 μM) alone or in combination with each other for 48 hours and cell viability was measured by MTT assay. Combination index (CI) values were calculated using the mean of three experiments for each combination and CompuSyn software. The CI values are illustrated in the heatmaps. CI > 1.3, antagonism; CI = 1.1–1.3, moderate antagonism; CI = 0.9–1.1, additive effect; CI = 0.8–0.9, slight synergism; CI = 0.6–0.8, moderate synergism; CI = 0.4–0.6, synergism; CI = 0.2–0.4, strong synergism. |

| 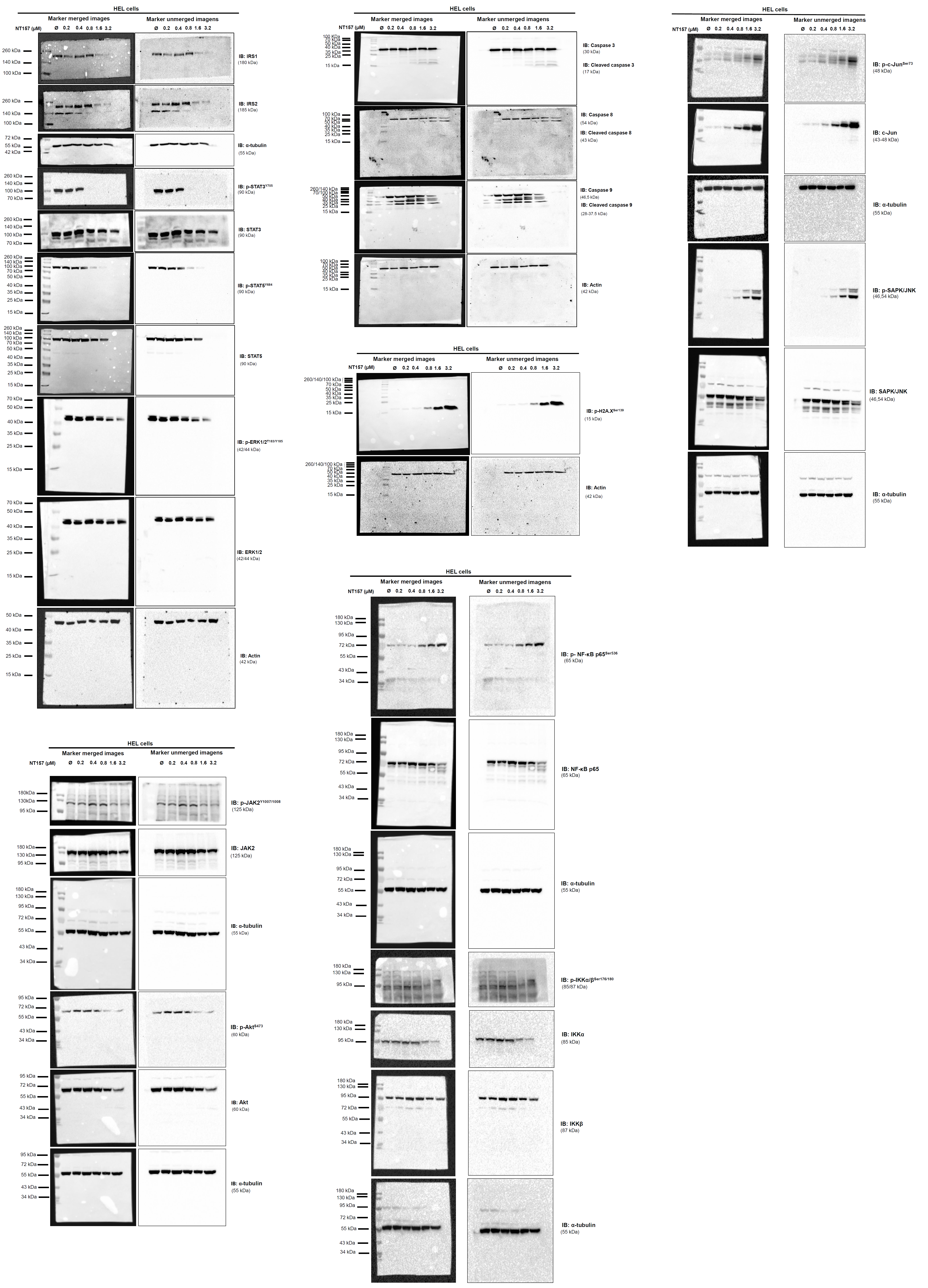 |
| --- |
| **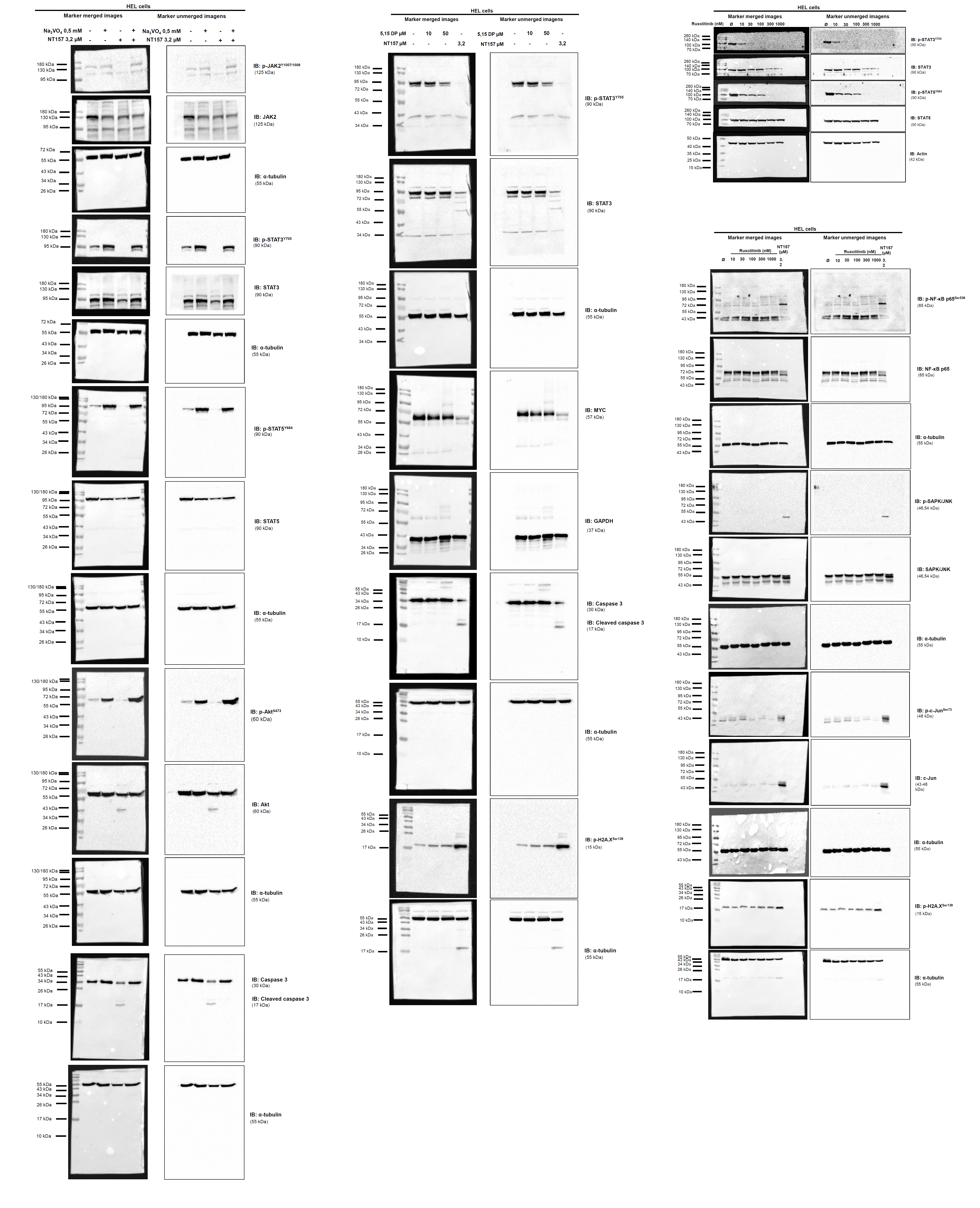** |
| **Supplementary Figure S4. Whole gel images of Western blot analysis**. Western blot analysis for protein phosphorylation and expression in total cell extracts from HEL cells treated with NT157 (Ø, 0.2, 0.4, 0.8, 1.6 and 3.2 μM) for 24 hours and submitted to IRS1/2, STAT3/5 and ERK proteins**,** caspases, and H2AX analysis. HEL cells treated with ruxolitinib (10, 30, 100, 300 and 1000 nM) for 24 hours and submitted to analysis of dose-response effects on STAT proteins. Membranes were reprobed with the antibody for the detection of the respective total protein or actin and developed with the SuperSignal™ West Dura Extended Duration Substrate system and a Gel Doc XR+ imaging system. The molecular weight of the ladder, antibodies, merged and unmerged images are indicated. |

**Supplementary Table S1.** Cell cycle raw data results from NT157 treatment

| **Cell cycle phase** | **NT157 (µM)** | | | |
| --- | --- | --- | --- | --- |
|  | **Ø** | **0.2** | **0.4** | **0.8** |
| **G_0_/G_1_ (% of cells)** |  |  |  |  |
| **Experiment #1** | 36.13 | 38.92 | 23.53 | 19.13 |
| **Experiment #2** | 30.39 | 31.16 | 27.46 | 17.55 |
| **Experiment #3** | 36.25 | 31.26 | 28.54 | 20.86 |
| **Mean, SD** | **34.26, 3.35** | **33.78, 4.45** | **26.51, 2.64** | **19.18, 1.66** |
|  |  |  |  |  |
| **S (% of cells)** |  |  |  |  |
| **Experiment #1** | 42.93 | 39.44 | 33.86 | 42.18 |
| **Experiment #2** | 48.99 | 47.37 | 40.99 | 35.96 |
| **Experiment #3** | 45.42 | 47.64 | 41.96 | 37.83 |
| **Mean, SD** | **45.78, 3.05** | **44.82, 4.66** | **38.94, 4.42** | **38.66, 3.19** |
|  |  |  |  |  |
| **G_2_/M** **(% of cells)** |  |  |  |  |
| **Experiment #1** | 20.94 | 21.64 | 42.61 | 38.69 |
| **Experiment #2** | 20.62 | 21.47 | 31.55 | 46.49 |
| **Experiment #3** | 18.34 | 21.09 | 29.50 | 41.31 |
| **Mean, SD** | **19.97, 1.42** | **21.40, 0.28** | **34.55, 7.05** | **42.16, 3.97** |

| **Supplementary Table S2. Human Oncogenes & Tumor Suppressor Genes investigated by PCR array** | | |
| --- | --- | --- |
| **Plate position** | **Gene** | **Fold change of control** |
| A12 | ***CCND1*** | 0,2404 |
| C05 | ***HGF*** | 0,4573 |
| B01 | ***CDH1*** | 0,4575 |
| E12 | ***RARA*** | 0,4755 |
| B05 | ***CDKN2B*** | 0,5346 |
| D01 | ***KIT*** | 0,61 |
| C02 | ***FHIT*** | 0,6176 |
| G09 | ***WT1*** | 0,6354 |
| E10 | ***PRKCA*** | 0,6371 |
| D11 | ***MYB*** | 0,6388 |
| E09 | ***PML*** | 0,6415 |
| E04 | ***NFKB1*** | 0,6456 |
| B04 | ***CDKN2A*** | 0,6603 |
| F10 | ***SH3PXD2A*** | 0,6617 |
| E05 | *NFKBIA* | 0,6787 |
| F08 | *S100A4* | 0,72 |
| C01 | *ETS1* | 0,7224 |
| B06 | *CDKN3* | 0,7299 |
| E01 | *MYCN* | 0,7514 |
| G03 | *TGFB1* | 0,7594 |
| D09 | *MLH1* | 0,7873 |
| F01 | *RASSF1* | 0,7889 |
| C12 | *JUND* | 0,7903 |
| B12 | *ESR1* | 0,7997 |
| A03 | *APC* | 0,818 |
| F05 | *ROS1* | 0,8187 |
| G01 | *STAT3* | 0,8238 |
| D10 | *MOS* | 0,8272 |
| A04 | *ATM* | 0,8343 |
| C09 | *JAK2* | 0,8419 |
| B02 | *CDK4* | 0,842 |
| A08 | *BCR* | 0,8604 |
| H05 | *ACTB* | 0,8628 |
| G11 | *XRCC1* | 0,8644 |
| H01 | *B2M* | 0,8724 |
| E08 | *PIK3CA* | 0,8783 |
| G05 | *TP53* | 0,8783 |
| G02 | *STK11* | 0,883 |
| G12 | *ZHX2* | 0,9164 |
| G08 | *VHL* | 0,9197 |
| A07 | *BCL2L1* | 0,9251 |
| G06 | *TP73* | 0,9258 |
| E03 | *NF2* | 0,9365 |
| E06 | *NRAS* | 0,9367 |
| D08 | *MGMT* | 0,9408 |
| B07 | *CTNNB1* | 0,9637 |
| F02 | *RB1* | 0,9703 |
| B10 | *ELK1* | 0,9771 |
| C11 | *JUNB* | 0,981 |
| C07 | *HRAS* | 0,9929 |
| A09 | *BRCA1* | 1,0223 |
| D06 | *MEN1* | 1,039 |
| F04 | *RET* | 1,0416 |
| A05 | *BAX* | 1,0566 |
| F09 | *SERPINB5* | 1,06 |
| H04 | *GAPDH* | 1,0823 |
| D12 | *MYC* | 1,0884 |
| G10 | *WWOX* | 1,0885 |
| H02 | *HPRT1* | 1,1002 |
| H03 | *RPL13A* | 1,1157 |
| A02 | *AKT1* | 1,1217 |
| D03 | *KRAS* | 1,1508 |
| H12 | *PPC* | 1,1599 |
| H07 | *RTC* | 1,1624 |
| H08 | *RTC* | 1,1624 |
| B11 | *ERBB2* | 1,1665 |
| G07 | *TSC1* | 1,1671 |
| E11 | *RAF1* | 1,1828 |
| F11 | *SMAD4* | 1,1837 |
| H10 | *PPC* | 1,1864 |
| B08 | *E2F1* | 1,1922 |
| H11 | *PPC* | 1,2019 |
| F03 | *REL* | 1,2039 |
| C08 | *IGF2R* | 1,2213 |
| G04 | *TNF* | 1,2563 |
| A01 | *ABL1* | 1,2631 |
| E02 | *NF1* | 1,3117 |
| H06 | *HGDC* | 1,3277 |
| H09 | *RTC* | 1,3291 |
| D04 | *MCL1* | 1,3385 |
| A11 | *CASP8* | 1,3491 |
| D05 | *MDM2* | 1,3669 |
| C06 | *HIC1* | 1,4396 |
| D02 | *KITLG* | 1,444 |
| F06 | *RUNX1* | 1,4542 |
| F12 | *SRC* | 1,4542 |
| A10 | *BRCA2* | 1,4946 |
| A06 | ***BCL2*** | 1,5472 |
| F07 | ***RUNX3*** | 1,5896 |
| E07 | ***PIK3C2A*** | 1,7171 |
| C03 | ***FOS*** | 1,9211 |
| C10 | ***JUN*** | 2,2852 |
| B09 | ***EGF*** | 2,653 |
| C04 | ***FOXD3*** | 3,4685 |
| D07 | ***MET*** | 5,4541 |
| B03 | ***CDKN1A*** | 9,9616 |

Samples submitted to NT157 treatment 0.8 µM for 16 hours related to untreated cells (control). Relative gene expression that presents 1.5 fold-change of control are highlighted in bold.

Supplementary Table S3. Cell cycle raw data results from combined treatment

| **Cell cycle phase** | **Monotherapy** | | | | **Combined** | |
| --- | --- | --- | --- | --- | --- | --- |
|  | **Ø** | **Rx 300 nM** | **NT157 0.4 µM** | **NT157 0.8**  **µM** | **Rx + NT157 0.4 µM** | **Rx + NT157 0.8 µM** |
| **G_0_/G_1_ (% of cells)** |  |  |  |  |  |  |
| **Experiment #1** | 48.12 | 49.37 | 35.47 | 6.72 | 37.44 | 29.93 |
| **Experiment #2** | 42.24 | 48.77 | 35.51 | 10.34 | 43.12 | 26.94 |
| **Experiment #3** | 46.17 | 53.79 | 26.80 | 18.01 | 41.36 | 21.75 |
| **Experiment #4** | 53.85 | 53.32 | 26.99 | 15.42 | 41.37 | 23.09 |
| **Mean**  **SD** | **47.59 4.83** | **51.32**  **2.61** | **31.19**  **4.96** | **12.62 5.06** | **40.82 2.40** | **25.43 3.72** |
|  |  |  |  |  |  |  |
| **S (% of cells)** |  |  |  |  |  |  |
| **Experiment #1** | 33.29 | 31.60 | 30.89 | 36.50 | 32.91 | 33.25 |
| **Experiment #2** | 32.56 | 32.21 | 31.96 | 33.69 | 32.54 | 38.97 |
| **Experiment #3** | 25.03 | 23.30 | 25.14 | 12.13 | 25.99 | 29.91 |
| **Experiment #4** | 25.64 | 25.32 | 30.59 | 27.75 | 30.19 | 29.60 |
| **Mean**  **SD** | **29.13 4.40** | **28.11 4.47** | **29.64 3.06** | **27.52 10.89** | **30.40**  **3.18** | **32.94**  **4.35** |
|  |  |  |  |  |  |  |
| **G_2_/M (% of cells)** |  |  |  |  |  |  |
| **Experiment #1** | 18.59 | 19.03 | 33.64 | 56.79 | 29.65 | 36.82 |
| **Experiment #2** | 25.20 | 19.02 | 32.53 | 55.97 | 24.35 | 34.09 |
| **Experiment #3** | 28.79 | 22.90 | 48.07 | 69.87 | 32.65 | 48.34 |
| **Experiment #4** | 20.51 | 21.36 | 42.42 | 56.83 | 28.44 | 47.31 |
| **Mean**  **SD** | **23.27 4.61** | **20.58 1.90** | **39.16**  **7.40** | **59.86 6.68** | **28.77 3.44** | **41.64 7.24** |

Supplementary Table S4. Antibodies against total and phosphorylated proteins evaluated by Western blot

| **Company** | **Antibody against** | **Catalog number** |
| --- | --- | --- |
| Abcam PLC  (Cambridge, UK) | c-Myc | ab32072 |
| Santa Cruz Biotechnology (Santa Cruz, USA) | IRS-2  STAT3  STAT5  Phospho-H2A.X^S139^  actin  α-tubulin | sc-390761  sc-7179  sc-835  sc-517348  sc-1616  sc-5286 |
| Cell Signaling Technology (Danvers, USA) | IRS-1  Phospho-JAK2^Y1007/1008^  JAK2  Phospho-STAT3^Y705^  Phospho-STAT5^Y694^  Phospho-Akt^S473^  Akt (pan)  NF-κB p65  Phospho -IKKα/β^S176/180^  IKKα  IKKβ  Phospho-SAPK/JNK^T183/Y185^  SAPK/JNK  Phospho-c-Jun  c-Jun  caspase 3  caspase 8  caspase 9  GAPDH | #3407  #3771  #3230  #9131  #9359  #9271  #4685  #4764  #2697  #2682  #8943  #9251  #9252  #3270  #9165  #9665  #9746  #9502  #2118 |
| Life Technologies  (Carlsbad, USA) | Phospho-ERK1/2^T183/Y185^  ERK1/2 | 700012  44654G |
| SABiosciences Corporation  (Frederick, USA) | Phospho- NF-κB p65^S536^ | FE-001 |

**Supplementary Table S5.** Sequence of primers used for PCR array validation

| **Gene** | **Specific primers (5’ - 3’)** | **Concentration (nM)** |
| --- | --- | --- |
| *CCND1* | | FW: CTG GGT GTC CTA CAA ATG | | --- | | RV: AGC GGT CCA GGT AGT TCA T | | 300 |
| MYB | | FW: CTC CGC CTA CAG CTC AAC TCC | | --- | | RV: TCC TTT ATT CGC TTT TCC TTC TCA | | 150 |
| WT1 | | FW: CGC TAT TCG CAA TCA GGG TTA | | --- | | RV: GGG CGT GTG ACC GTA GCT | | 300 |
| *CDKN1A* | | FW: TGT CAC TGT CTT GTA CCC TTG T | | --- | | RV: GCC GGC GTT TGG AGT GGT AG | | 300 |
| JUN | | FW: CAG GTG GCA CAG CTT AAA CA | | --- | | RV: GTT TGC AAC TGC TGC GTT AG | | 150 |
| FOS | | FW: AGA ATC CGA AGG GAA AGG AA | | --- | | RV: CTT CTC CTT CAG CAG GTT GG | | 300 |
| *NFKB1* | FW: GGC AGC ACT ACT TCT TGA CC  RV: CAG CAA ACA TGG CAG GCT AT | 300 |
| *HPRT1* | FW: GAA CGT CTT GCT CGA GAT GTG  RV: TCC AGC AGG TCA GCA AAG AAT | 150 |
| *ACTB* | FW: AGG CCA ACC GCG AGA AG  RV: ACA GCC TGG ATA GCA ACG TAC A | 300 |
| *GAPDH* | FW: GGA GCG AGA TCC CTC CAA AAT  RV: GGC TGT TGT CAT ACT TCT CAT GG | 300 |
