# Supplementary figures and images for: NT157 has antineoplastic effects and inhibits IRS1/2 and STAT3/5 in JAK2V617F-positive myeloproliferative neoplasm cells
Source: Signal Transduct Target Ther. 2020 Jan 24;5:5. doi: 10.1038/s41392-019-0102-5 (PMC6978524; doi:10.1038/s41392-019-0102-5)

Whole Western blot gels for review

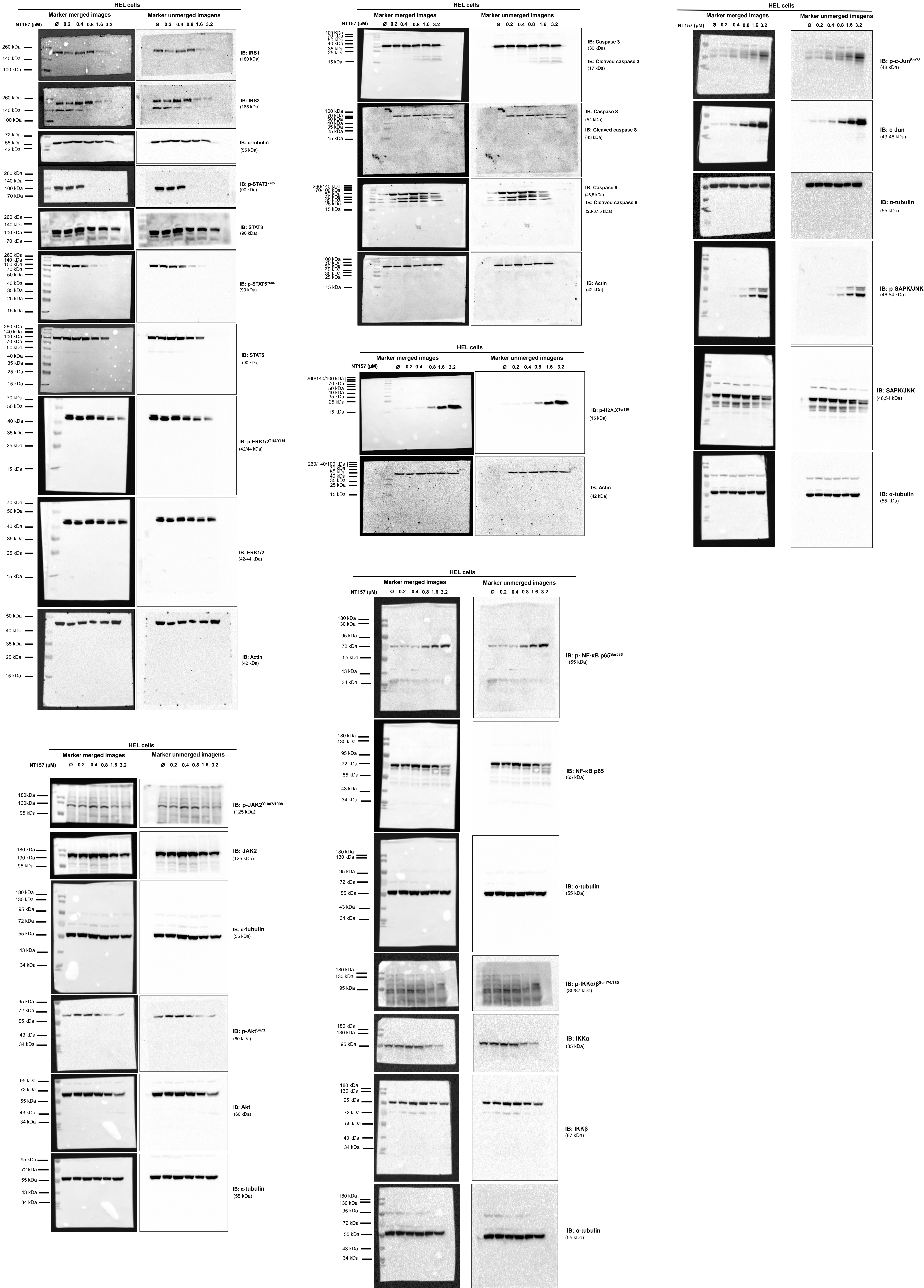

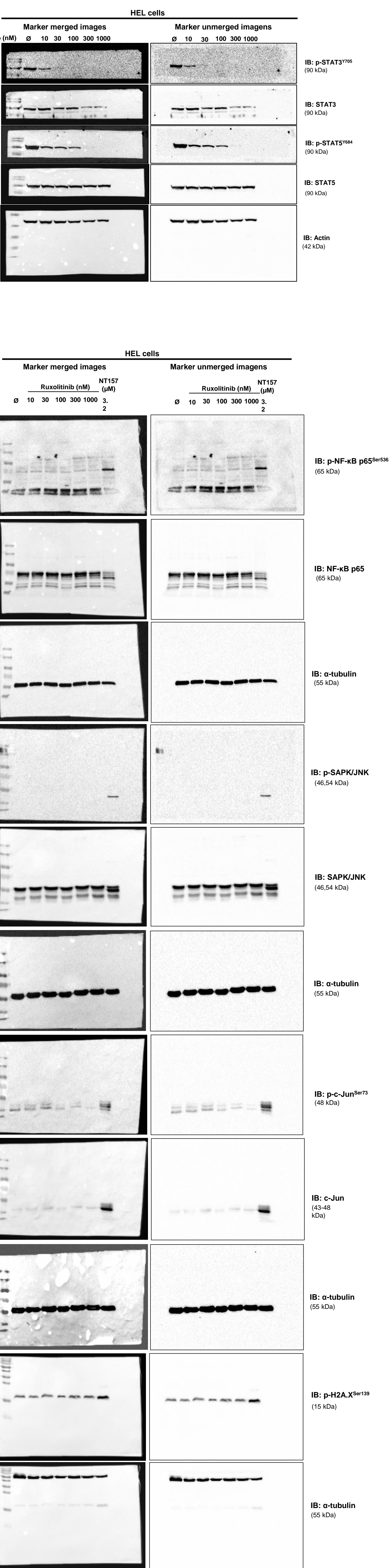

Supplement: Supplementary file 2 — Dataset 1 [file 41392_2019_102_MOESM2_ESM.pdf]
